# δ-Catenin promotes prostate cancer cell growth and progression by altering cell cycle and survival gene profiles

**DOI:** 10.1186/1476-4598-8-19

**Published:** 2009-03-10

**Authors:** Yan Zeng, Agustin Abdallah, Jian-Ping Lu, Tao Wang, Yan-Hua Chen, David M Terrian, Kwonseop Kim, Qun Lu

**Affiliations:** 1 Department of Anatomy and Cell Biology, Brody School of Medicine, East Carolina University, Greenville, NC 27858, USA; 2 Leo Jenkins Cancer Center, Brody School of Medicine, East Carolina University, Greenville, NC 27858, USA; 3 Department of Surgery, Beijing Capital Medical University, Beijing, PR China; 4 College of Pharmacy, Chonnam National University, Gwangju, Republic of Korea

## Abstract

**Background:**

δ-Catenin is a unique member of β-catenin/armadillo domain superfamily proteins and its primary expression is restricted to the brain. However, δ-catenin is upregulated in human prostatic adenocarcinomas, although the effects of δ-catenin overexpression in prostate cancer are unclear. We hypothesized that δ-catenin plays a direct role in prostate cancer progression by altering gene profiles of cell cycle regulation and cell survival.

**Results:**

We employed gene transfection and small interfering RNA to demonstrate that increased δ-catenin expression promoted, whereas its knockdown suppressed prostate cancer cell viability. δ-Catenin promoted prostate cancer cell colony formation in soft agar as well as tumor xenograft growth in nude mice. Deletion of either the amino-terminal or carboxyl-terminal sequences outside the armadillo domains abolished the tumor promoting effects of δ-catenin. Quantitative RT^2 ^Profiler™ PCR Arrays demonstrated gene alterations involved in cell cycle and survival regulation. δ-Catenin overexpression upregulated cyclin D1 and cdc34, increased phosphorylated histone-H3, and promoted the entry of mitosis. In addition, δ-catenin overexpression resulted in increased expression of cell survival genes Bcl-2 and survivin while reducing the cell cycle inhibitor p21^Cip1^.

**Conclusion:**

Taken together, our studies suggest that at least one consequence of an increased expression of δ-catenin in human prostate cancer is the alteration of cell cycle and survival gene profiles, thereby promoting tumor progression.

## Background

Tumor progression is the result of loss of balance in cellular functions including cell growth, adhesion, division and apoptosis. Among the many oncogenes and tumor suppressors, cell-cell junction associated proteins, such as β-catenin and adenomatous polyposis coli (APC), contribute to cancer development by disrupting the E-cadherin based cell-cell junction, as well as interfering with cell proliferation, altering karyotype, and reducing apoptosis [1,2].

δ-Catenin, or NPRAP (neural plakophilin related armadillo protein)/Neurojungin, is an adhesive junction associated protein [3,4], which was initially identified as a neural specific protein [5,6]. δ-Catenin belongs to the p120^ctn ^subgroup in the armadillo/β-catenin superfamily [5,7]. While β-catenin and p120^ctn ^are ubiquitously expressed in the body, δ-catenin distribution is principally restricted to the brain in healthy individuals. However, it has become increasingly clear that δ-catenin is expressed in a variety of cancers of peripheral tissues, including breast, prostate, and esophageal tumors [8,9]. Recently, we showed that δ-catenin is upregulated in over 80% of prostatic adenocarcinomas, and its expression is correlated with increasing Gleason scores [9]. An increased expression of δ-catenin is accompanied by reduced E-cadherin and p120^ctn ^in primary prostatic adenocarcinomas, and the forced overexpression of δ-catenin in cultured prostate cancer cells can induce the redistribution of E-cadherin and p120^ctn ^[9]. While it is established that cell-cell junction proteins, such as E-cadherin, β-catenin, APC, and p120^ctn^, are involved in cell adhesion and motility as well as cancer cell growth [1,2,10,11], it is not clear whether δ-catenin overexpression exerts any effects on prostate cancer cells.

In this study, we tested the hypothesis that δ-catenin plays a direct role in prostate cancer cell growth by altering gene profiles of cell cycle regulation and cell survival. We demonstrated, for the first time, that δ-catenin overexpression promotes anchorage-independent prostate cancer cell growth and tumor xenografts in nude mice. We determined that the ability of δ-catenin overexpression to promote prostate tumor xenograft growth is dependent on the amino- (NH_2_) and carboxyl- (COOH) terminal sequences flanking the armadillo repeat domains. In addition, quantitative RT^2 ^Profiler™ PCR arrays revealed a wide range of gene alterations involved in cell cycle and survival regulation. These findings support the notion that at least one consequence of an increased δ-catenin expression in prostate cancer development is the alteration of cell cycle and survival gene profiles, thereby promoting tumor progression.

## Results

### δ-Catenin overexpression promotes prostate cancer cell growth in culture

Our previous studies showed that δ-catenin expression is very weak in normal prostatic glandular epithelial cells but is remarkably increased in prostatic adenocarcinoma [9]. Screening for prostate cancer cell lines overexpressing δ-catenin, we found that δ-catenin expression was moderately increased in CWR22Rv-1 and PC-3 cells when compared to non-cancer prostate epithelial cells PZ-HPV-7 but remained very low in LNCaP and DU145 cells [12]. Therefore, CWR22Rv-1, derived from a recurrent human prostate cancer xenograft [13,14], and PC-3 cells, derived from a bone metastasis of prostatic adenocarcinoma [15], were chosen to test the hypothesis that the increase or decrease in δ-catenin expression affect prostate cancer cell growth, respectively.

**Figure 1:**
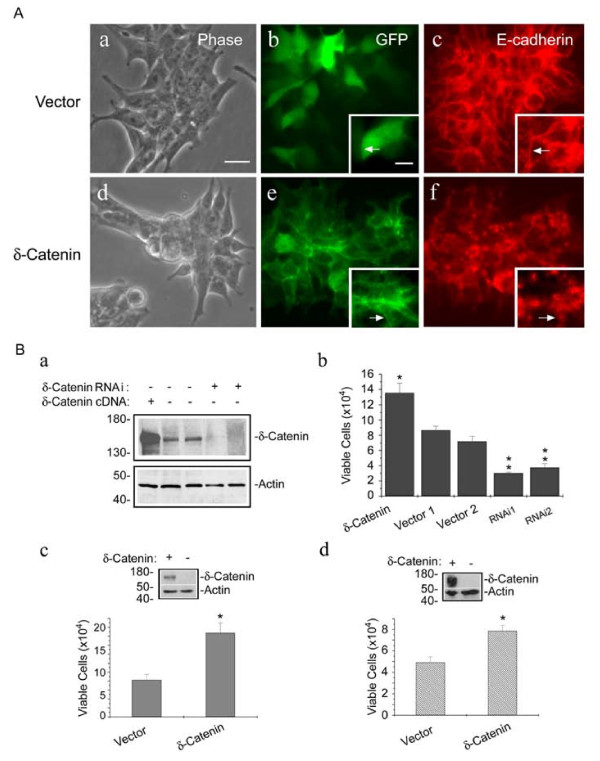
**δ-Catenin expression is important for viable prostate cancer cell growth**. A. Establishment of stable CWR22Rv-1 cells overexpressing δ-catenin and its effects on epithelial cell morphology. CWR22Rv-1 cells, showing epithelial morphology (a) when transfected with *pEGFP *as vector control (b), display expression of E-cadherin at the cell-cell junction (c). δ-Catenin overexpressing CWR22Rv-1 cells (e) interfere with the epithelial monolayer (d) and disrupt E-cadherin expression at the cell-cell junction (f). Bar, 30 μm. Inserts: selective higher magnification images for (b), (c), (e) and (f), respectively. Bar, 25 μm. B. δ-Catenin overexpression promotes, while its knockdown suppresses prostate cancer CWR22Rv-1 cell growth. a. Western blot analysis shows that the increased expression of δ-catenin in cells transfected with δ-*catenin *cDNA and reduced expression of δ-catenin in cells transfected with δ-*catenin *shRNA. Anti-actin staining is used as a loading control, and the molecular weight markers (kDa) are on the left. b. δ-*Catenin *shRNA transfection reduces viable cell numbers while δ-catenin overexpression by δ-*catenin *cDNA transfection increases viable cell numbers. Vector 1 and 2: *pRS-GFP and pEGFP*, respectively. shRNA 1 and shRNA 2: shRNA against δ-*catenin *sequences 1 and 2, * P < 0.05. c and d. δ-Catenin overexpression promotes cancer cell growth in PC-3 (c) and NCI-H1299 (d) cells. Inserts: Western blots showing PC-3 (c) and NCI-H1299 (d) cells with (+) or without (-) stable δ-catenin overexpression. Anti-actin staining is used as a loading control, and the molecular weight markers (kDa) are on the left.

We transfected δ-*catenin *cDNA with EGFP fusion into CWR22Rv-1 cells (Fig 1A). The stable cell lines were established by G418 selection followed by cell sorting to enrich for GFP emitting cell populations. This method allowed for the establishment of cell culture that contained almost 100% δ-catenin overexpressing cells. While vector transfected cells showed clear monolayer cell morphology (Fig 1A, a), δ-catenin overexpressing cells tended to form clusters (Fig 1A, d). This result was reminiscent of the disruption of cell monolayer morphology in MDCK kidney epithelial cells overexpressing δ-catenin [3]. Vector transfected cells, with GFP as the transfection marker uniformly distributed in the cells (Fig 1A, b; see insert), displayed E-cadherin at the cell-cell junction (Fig 1A, c; see insert). However, δ-catenin overexpression at the cell-cell junction (Fig 1A, e; see insert) showed disrupted E-cadherin distribution (Fig 1A, f; see insert).

A number of studies showed that disrupted cell-cell junction can alter cell growth [10,11]. To investigate the effects of δ-catenin expression in prostate cancer cells, we examined the growth property of CWR22Rv-1 cells stably overexpressing δ-catenin or stably suppressing δ-catenin expression (Fig 1B). To determine if the endogenous δ-catenin expression plays an important role in cell growth, stable cell lines expressing small hairpin RNAs (shRNAs) specific for δ-*catenin *gene were established and confirmed by Western blots (Fig 1B, a). Compared with vector transfected cells, δ-catenin overexpression showed a significant increase in cell numbers (Fig 1B, b, compare δ-catenin with vector). Compared with vector transfected cells, two independent shRNAs against different δ-*catenin *sequences reduced viable cell numbers (Fig 1B, b, compare vector 1 and 2 with shRNA 1 and 2).

To determine if the growth promoting effects of δ-catenin expression also applies to other cancer cell types, we examined PC-3 prostate cancer cells and NCI-H1299 cells derived from a human lung carcinoma [16]. Similarly, viable cell numbers were significantly increased in PC-3 and NCI-H1299 cells stably overexpressing δ-catenin (Fig 1B, c and 1B, d). These observations indicated that δ-catenin expression is important for cancer cell growth in culture.

### δ-Catenin promotes the anchorage-independent growth of CWR22Rv-1 cells in soft agar and tumor xenografts in nude mice

**Figure 2:**
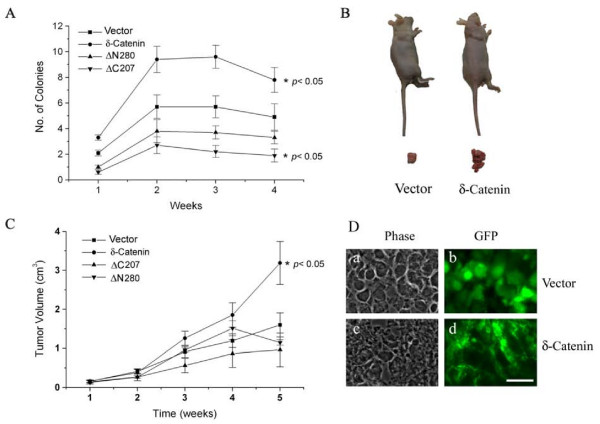
**δ-Catenin expression mediated anchorage-independent prostate cancer cell colony formation and tumor xenograft growth replies on the NH_2_- and COOH-terminal sequences outside armadillo domains**. A. Soft agar assays showing that full length δ-catenin, but not its NH_2_- or COOH-terminal truncation mutants, promotes CWR22Rv-1 cell colony formation in vitro. Stable cells expressing vector control, full-length δ-catenin, ΔN280 and ΔC207 were plated in soft agar. Colonies were counted under the phase contrast light microscope in 1, 2, 3, and 4 weeks after they were plated. Results were derived from five independent experiments, each in duplicate. * P < 0.05. B. δ-Catenin overexpression promotes CWR22Rv-1 cells to grow tumors in nude mice. Shown here is a representative pair of tumor bearing mice expressing control vector and δ-catenin, respectively (n = 19). C. Full-length δ-catenin, but not its NH_2_- or COOH-terminal truncation mutants, promotes tumor xenograft growth in nude mice. Male athymic nude mice were inoculated subcutaneously with either the vector, full-length δ-catenin, ΔC207 or ΔN280 and were then allowed to grow for 5 weeks. Each week, the tumor volumes were measured and compared to each other. * P < 0.05. D. Fluorescent light microscopy showing the tumor cell morphology (a and c) and GFP positive CWR22Rv-1 cells expressing vector alone (b) and overexpressing δ-catenin (d). Bar, 50 μm.

To investigate whether δ-catenin overexpression promotes the anchorage-independent growth of prostate cancer cells, we performed soft agar assays. Following the plating of δ-catenin transfected and vector transfected CWR22Rv-1 cells in 0.6% top agar, cell colonies were scored after 1, 2, 3 and 4 weeks of incubation, respectively. The colony number of δ-catenin overexpressing cells increased more than 2 fold when compared to that of vector transfected cells during 1, 2 and 3 weeks (Fig 2A, compare δ-catenin with vector). The colony number as well as the colony size was decreased in both δ-catenin overexpressing cells and control cells by the 4^th ^week, consistent with the literature that colony formation decreases after prolonged incubation [17]. This analysis revealed that δ-catenin overexpression promoted colony formation in soft agar.

To determine whether δ-catenin overexpression promotes tumor growth in vivo, we examined the effect of δ-catenin expression on the growth of CWR22Rv-1 tumor xenograft in nude mice. δ-Catenin overexpressing cells produced significantly larger tumors than that of the vector transfected cells (Fig 2B, n = 19). The volume of tumors derived from δ-catenin transfected cells increased steadily two weeks after inoculation and was nearly 2 fold when compared to that of tumors from control cells (Fig 2C, compare δ-catenin with vector). Similar results were observed when PC-3 and NCI-H1299 cells overexpressing δ-catenin were used for inoculating the nude mice (data not shown). Examination of tumor histology under fluorescent light microscopy showed that the majority of tumor volume contained green fluorescent cells, confirming that the tumors were indeed the result of proliferation and growth of inoculated CWR22Rv-1 vector transfected cells (Fig 2D, a and 2D, b) or cells overexpressing δ-catenin (Fig 2D, c and 2D, d), respectively.

### The tumor promoting effects of δ-catenin relies on the NH_2_- and COOH-terminal sequences outside the armadillo domains

**Figure 3:**
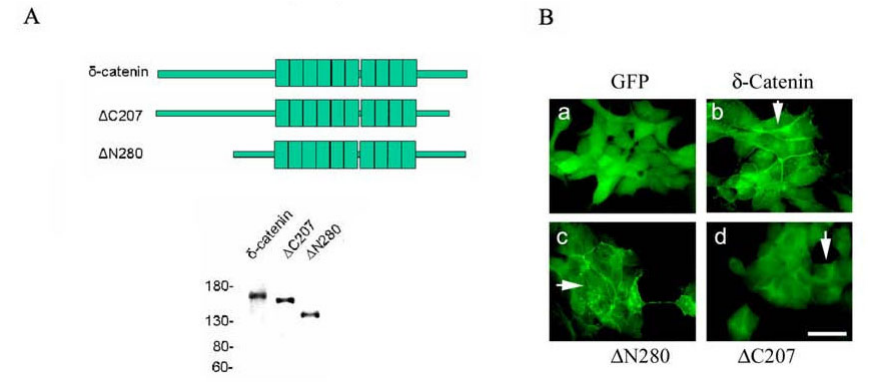
**A. Establishment of stable CWR22Rv-1 cells overexpressing full-length, ΔN280 and ΔC207 δ-*catenin *cDNAs**. Top panel, schematic illustration of full-length, ΔN280 and ΔC207 δ-*catenin *cDNAs. Bottom panel, western blots showing the stable expression of full-length, ΔN280 and ΔC207 δ-catenin protein in CWR22Rv-1 cells. Molecular weight markers are shown on the left. B. GFP fluorescent images of CWR22Rv-1 cells stably expressing vector (a), fill-length δ-catenin (b, arrow points to cell-cell junction), ΔN280 (c, arrow points to cell-cell junction) and ΔC207 (d, arrow points to cytoplasmic distribution). Bar, 50 μm.

While the armadillo domains of δ-catenin showed considerable homology to other β-catenin/armadillo family proteins, the NH_2_- and COOH-terminal sequences flanking the armadillo repeat units are quite different among the different members of the family [5,7]. Therefore, we transfected δ-*catenin *cDNA with NH_2_-terminal (ΔN280) or COOH-terminal (ΔC207) deletions (Fig 3A, top panel) and established the respective cell lines showing stable expression (Fig 3A, bottom panel). While GFP protein was diffusely distributed in the nucleus and cytoplasm in CWR22Rv-1 cells (Fig 3B, a), full-length δ-catenin (Fig 3B, b), arrow) and ΔN280 (Fig 3B, c, arrow) were similarly localized at the cell-cell junction. However, ΔC207 was not associated with cell-cell junctions and remained mainly cytoplasmic (Fig 3B, d, arrow). This result was consistent with ΔC207 distribution in NIH3T3 cells and in neurons [18,19]. When their growth in soft agar was compared, it became clear that compared to full-length δ-catenin, both ΔN280 and ΔC207 did not promote colony formation in soft agar (Fig 2A). In fact, ΔC207 showed significant inhibition of forming cell colonies as compared to vector transfected cells (*p *< 0.05). Similarly, ΔN280 and ΔC207 did not show tumor promoting effects in nude mice (Fig 2C), although ΔN280 and ΔC207 did not show statistically significant inhibition of tumorigenesis when compared to vector control. These results supported the notion that δ-catenin sequences outside the armadillo domains are important for its tumor promoting activity.

### δ-Catenin promotes the entry of mitosis in CWR22Rv-1 cells

**Figure 4:**
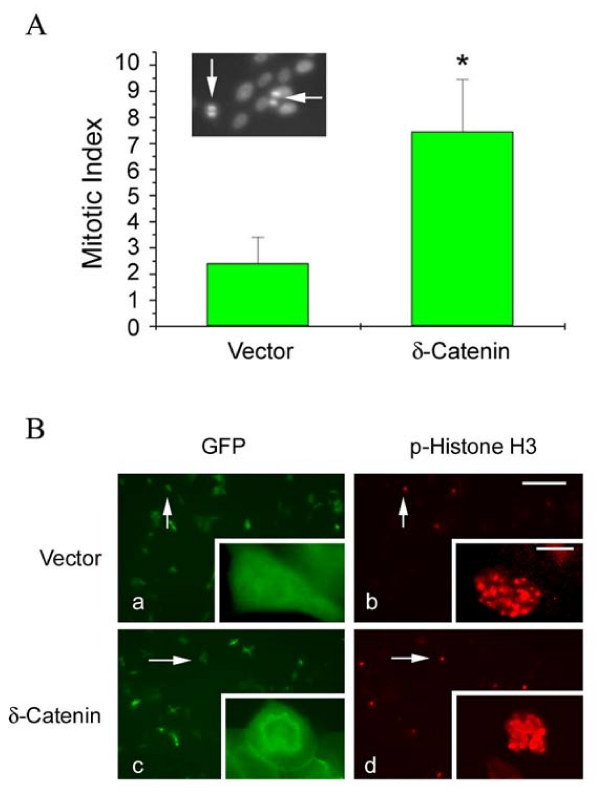
**δ-Catenin expression promotes the entry of mitosis**. A. Mitotic index of cells expressing vector alone as a control and cells overexpressing δ-catenin. Mitotic index is given as the percentage of cells entering mitosis determined by Hoechst staining. Insert: mitotic cells (arrows) showing strong Hoechst staining. B. Immunofluorescent light microscopy showing increased mitotic activity in δ-catenin expressing cells (c and d) in comparison to control vector transfected cells (a and b). Mitotic activity is determined as the number of GFP positive, transfected cells (a and c) intensely reactive with anti-phosphorylated histone H3 (p-Histone H3, b and d). Arrows: examples of GFP positive, transfected cells immunoreactive for phosphorylated histone H3. Bar, 100 μm; Bar in the insert, 5 μm.

To investigate if the effect of δ-catenin overexpression on the increase in viable cell number was to increase mitosis, we conducted experiments to compare the mitotic index of δ-catenin expressing cells with that of vector transfected cells. Cells entering mitosis show intense chromosomal staining with Hoechst 33258 dye highlighting different phases of cell division (Fig 4A, insert). Percentages of cells showing clear mitotic figures over the total number of cells were counted as mitotic index. This analysis showed that CWR22Rv-1 cells overexpressing δ-catenin had a significantly higher mitotic index (Fig 4A), consistent with increased entry to mitosis.

To independently confirm the increased mitotic index in δ-catenin overepressing cells, we analyzed phosphorylated histone-H3 immunolocalization (Fig 4B). Histone-3 is increasingly phosphorylated when cells pass G2 phase and enter mitosis. Control, vector transfected CWR22Rv-1 cells showed 3 ± 0.20% cells with phosphorylated hisotne-H3 immuoreactivity (Fig 4B, a and 4B, b, arrows), whereas δ-catenin overexpressing cells showed a 7 ± 0.32% phosphorylated histone-H3 activity (Fig 4B, c and 4B, b, arrows), supporting the notion that more δ-catenin overexpressing cells entered mitosis.

### δ-Catenin overexpression alters gene profiles of cell cycle and survival

**Figure 5:**
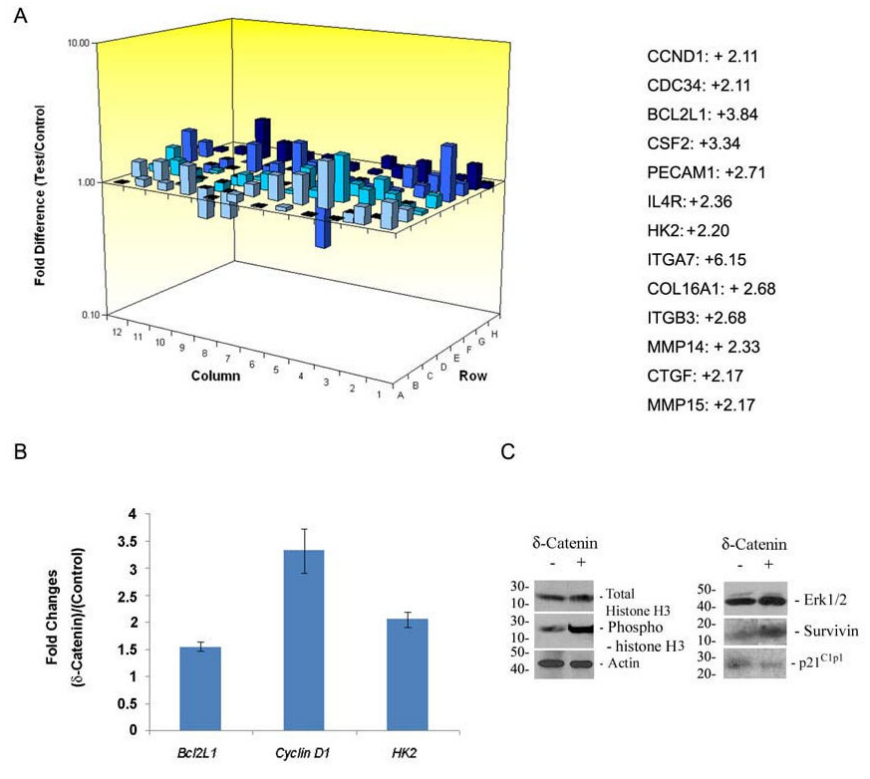
**Changes in gene profiles of CWR22Rv-1 cells expressing δ-catenin when compared to that of cells expressing vector alone**. A. RT^2 ^Profiler™ PCR Array. CWR22Rv-1 cells with or without δ-catenin overexpression were subject to RT^2 ^Profiler™ PCR arrays of apoptosis and cell cycle. Left panel shows the schematic illustration of one of the array outcomes. Right panel shows selected genes revealing over 2-folds upregulation. B. Selective, single real-time PCR analyses to compare RNA expression of *cyclin D1*, *Bcl2L1 *and *HK2 *in CWR22Rv-1 cells with or without δ-*catenin *overexpression. C. Changes in protein expression in CWR22Rv-1 cells with (+) or without (-) δ-catenin overexpression. Cells were lysed and proteins were separated by SDS-PAGE followed by Western blots using antibodies against proteins indicated on the right. After the blots were exposed using chemiluminence, the same blot was re-probed using mouse anti-actin to demonstrate protein loading control. Molecular weight markers in kDa are indicated on the left.

To further explore the potential molecular mechanisms underlying the δ-catenin mediated increase in prostate cancer cell viability, we investigated the gene expression patterns of cell cycle and survival. Compared to vector transfected cells, δ-catenin overexpression led to changes in a number of genes in these pathways. Notably, cell cycle regulatory genes such as cyclin D1 (CCND1) and cdc34 (CDC34), activation of which promotes G1 to S transition [20], showed more than a 2-fold increase (Fig 5A). Bcl2L1 (BCL2L1), the long isoform of Bcl2 involved in anti-apoptotic activation [21], increased by 3.84 fold. CSF2 (colony stimulating factor 2)/GM-CSF (granulate-macrophage colony stimulating factor), involved in tumor metastasis to the bone [22], showed a 3.34-fold increase (Fig 5A). ITGA7 (integrin α7) increased by 6.15 fold whereas other cell adhesion and matrix genes such as COL16A1 (collagen 16), ITGB3 (integrin β3), MMP14, MMP15 and CTGF (connective tissue growth factor), each showed increases over 2 fold. One unique biochemical property within prostate epithelial cells is their dependence on glycolysis for energy production. HK2 (Hexokinase 2), involved in the increased rate of glycolysis seen in rapidly growing cancer cells [23], was increased 2.2-fold (Fig 5A). To confirm the validity of RT^2 ^Profiler™ PCR Array, we selected *cyclin D1*, *Bcl2L1*, and *HK2 *genes to further study their changes in expression by real-time PCR (Fig 5B). *Cyclin D1 *showed an over 3-fold increase in expression, *Bcl2L1 *expression increased 1.5-fold, and *HK2 *turned in an over 2-fold increase in expression, in line with the array results (Fig 5A).

We also selected several protein markers to evaluate their potential involvement in the regulation of cell growth by δ-catenin overexpression. For example, while the total expression level of histone-H3 was similar in both vector and δ-catenin transfected cells (Fig 5C), histone-H3 phosphorylation was clearly elevated in δ-catenin overexpressing cells when compared to control cells, which supports the immunofluorescent light microscopic observations of increased mitotic activity in δ-catenin overexpressing cells (Fig 4B). Although Erk1/2 expression did not show any changes, the expression of survivin, a member of the inhibitor-of-apoptosis family known to be overexpressed by cancer cells, was increased in δ-catenin overexpressing CWR22Rv-1 cells when compared to control cells (Fig 5C). Cell cycle inhibitor p21^Cip1 ^is a downstream effector of p53 mediated cell cycle interruption [24,25]. However, p21^Cip1 ^expression in δ-catenin overexpressing cells was reduced when compared to vector transfected cells (Fig 5C). These results indicated that the effects of δ-catenin on cell viability could also be due to reduced cell death, in addition to an increased mitotic activity.

## Discussion

β-Catenin/armadillo superfamily proteins are expressed in cells of all major tissue types including epithelial cells. They play important roles in cancer with some of its members as oncogenes while others act as tumor suppressors [1,2]. δ-Catenin (gene designation as *CTNND2*), however, is a unique member of the family because it is primarily expressed in the central nervous system of normal individuals. However, δ-catenin is now well established as being overexpressed in prostate cancer [9,26].

The frequently increased expression of the neuronal protein δ-catenin in peripheral prostate cancer tissue raised important questions such as what mechanisms are in place in cancer to result in a high level of δ-catenin expression. δ-*Catenin *gene amplification was observed in cervical cancer [27] and bladder cancer [28]. Transcription factor Pax6 was found to play an important role for regulation of δ-catenin expression in developing eye and central nervous system [29]. Recently, we showed that the ectopic overexpression of E2F1 and Pax6 positively upregulates δ-catenin expression in prostate cancer cells [12]. Furthermore, increased translation efficiency by somatic mutations in the 5'-untranslated region of δ-*catenin *was observed in prostate cancer patients [30], further supporting the hypothesis that cancer cells implement multiple mechanisms to upregulate δ-catenin expression to advance tumor progression. In this study, we provided the first evidence that δ-catenin is capable of promoting the expansion of prostate cancer cells, altering gene profiles of prostate cancer cell cycle regulation and survival.

Regarding cell proliferation and colonization, earlier studies evaluating MDCK cells transfected with δ-catenin showed that there were no significant differences between MDCK cells expressing δ-catenin and control cells [3]. Interestingly, ectopic expression of δ-catenin in NIH3T3 fibroblast cells inhibited cell division and induced cellular processes with branches [18]. However, δ-catenin overexpression in pheochromocytoma (PC12) cells increased cell proliferation and promoted neurite outgrowth when treated with nerve growth factor [31]. These studies, in addition to our current findings, suggest that δ-catenin effects on cell growth are context dependent. It is possible that preneoplastic cells may not tolerate high levels of stable δ-catenin expression, such as in MDCK cells [3], NIH3T3 cells [18], and mammary epithelial cells [32]. We have also failed to develop stable cell lines using PZ-HPV-7 (non-cancer human prostate epithelial origin) and NL20 (non-cancer human lung epithelial origin) cells (Zeng and Lu, unpublished data). However, in tumor cells, such as PC12, CWR22Rv-1, PC-3 and NCI-H1299, stable cell lines with δ-catenin overexpression were not only successfully produced, but also showed increased cell viability, suggesting that δ-catenin does not transform normal cells but promotes cancer cell expansion.

The armadillo repeating units reveal the most significant homology among β-catenin superfamily proteins, while the sequences flanking the armadillo domain are quite variable [5,7]. This feature is consistent with the hypothesis that sequences outside the armadillo domains play important regulatory roles to characterize each different member. δ-Catenin interacts with classical cadherins through the armadillo domains, and the NH_2_- or COOH-terminal sequences on their own do not localize to cell-cell junctions [3]. In addition, the deletion of COOH-terminal 207 amino acids abolished the δ-catenin mediated process extension in NIH3T3 cells and compromised the roles of δ-catenin in promoting dendrite outgrowth in neurons [18,33,34]. Removing the NH_2_-terminal 280 amino acids also remarkably altered the effects of δ-catenin on 3T3 cell morphology [18]. In our present study, we showed that while the full-length δ-catenin promoted prostate cancer cell colony formation in soft agar and tumor xenograft growth in nude mice, δ-catenin with the deletion of either NH_2_-terminal 280 amino acids or COOH-terminal 207 amino acids lost its ability to promote tumor development. These studies underscore the importance of these sequences outside the armadillo domain to the functions of δ-catenin in cancer.

Gene profiling can provide initial indications of what may be the potential molecular pathways that δ-catenin employs to contribute to tumor progression. We identified a number of genes that displayed changes in expression when δ-catenin is overexpressed in prostate cancer cells. Several of these genes, such as cyclin D1, cdc34, Bcl2L1, and HK2, are especially interesting. Both cyclin D1 and cdc34 are involved in G1 to S transition, and Bcl2L1 is a long isoform of Bcl2 which protects cells from undergoing apoptosis [21]. HK2 phosphorylates glucose to produce glucose-6-phosphate, thus committing glucose to the glycolytic pathway. Expression of HK2 has been indicated in rapidly growing cancer cells [35,36]. Nevertheless, we still do not know how an increased δ-catenin expression affects these pathways in prostate cancer development. Our future studies will dissect the signaling events to determine the mechanisms by which δ-catenin employs to promote prostate cancer cell growth and tumor progression.

## Conclusion

This study is the first to demonstrate the effects of δ-catenin overexpression in prostate cancer cells. We show that these effects rely on the δ-catenin domains outside armadillo repeating sequences, providing future direction for investigating the molecular basis of δ-catenin mediated prostate cancer development. Our studies suggest that an increased expression of δ-catenin in human prostate cancer permits the alteration of cell cycle and survival gene profiles, which may advance tumor progression.

## Methods

### Materials

Mouse anti-δ-catenin and p21^Cip1 ^were from BD Biosciences (Palo Alto, CA). Rabbit anti-cyclin D1 were from Santa Cruz Biotech (Santa Cruz, CA). Mouse anti-actin, histone-H3, and rabbit anti-Erk1/2 and survivin were from Cell Signaling (Boston, MA). Rabbit anti-phosphorylated histone-H3 (Ser 10) was obtained from Upstate Biotech (Lake Placid, NY). Unless otherwise indicated all chemicals were from Sigma (St. Louis, MO).

### Cell Culture and Transfection

CWR22Rv-1 and PC-3, as well as NCI-H1299, were obtained from ATCC and cultured in RPMI 1640 medium (Invitrogen, Carlsbad, CA) supplemented with 10% fetal bovine serum (FBS). All cultures were maintained at 37°C with 5% CO_2 _until they were used for further experimental analyses.

To produce stable δ-catenin overexpressing cell lines, full length, NH_2_-terminal deleted (ΔN280), or COOH-terminal deleted (ΔC207) δ-*catenin *cDNA with or without *pEGFP *fusion [18] was transfected using FuGENE 6 (Roche Scientific, Gaithersburg, MD). CWR22Rv-1, PC-3 and NCI-H1299 cells transfected with *pEGFP *(Clontech, Palo Alto, CA) were used as a vector control. Stable cell lines transfected with untagged δ-catenin were established by selection using 250 μg/ml G418. For selection of *pEGFP-δ-catenin *transfected cells, cells were first selected in G418 containing medium. Then, *pEGFP-δ-catenin *trasnfected cells were further selected by GFP-based cell sorting using a FACS Vantage (BD Biosciences). The stable cell lines were maintained in RPMI 1640 medium containing G418.

### shRNA and Western Blot Analysis

δ-*Catenin *gene specific shRNA constructs (gene sequence accession number NM 001332) used in this study were from Origene Technologies, Inc (Rockville, MD). They were as follows: shRNA1, 5'-ggatggagtaggacctcttccagactgtg-3', shRNA2, 5'-ctcacgctttgtttactctcttcatccgt-3', and a negative control shRNA *pRS-GFP *plasmid. CWR22Rv-1 cells were transfected with shRNA specific to δ-*catenin *gene using Lipofectamine 2000 (Invitrogen) and co-transfected with *pGFP*. Following transfection, the medium was replaced by RPMI 1640 with G418 for selection. The G418 resistant cells were further sorted using GFP as a marker to enrich for shRNA expressing cells.

To determine δ-catenin knockdown efficiency, cultured cells were lysed in 10 mM HEPES, pH 7.3, 150 mM NaCl, 2 mM EDTA, 1% Triton X-100, 0.5% deoxycholate, 0.2% SDS with protease inhibitor cocktails (Radioimmunoprecipitation buffer, RIPA). Insoluble materials were removed by centrifugation. The lysates were mixed with sample buffer and equal protein amounts were loaded onto gels for SDS-PAGE and Western blot analysis. After proteins were transferred to nitrocellulose membranes (PGC Scientifics, Frederick, MD), anti-δ-catenin and anti-actin immunoreactivities were revealed by antibody immunoblotting using enhanced chemiluminescence (Amersham Life Science, Piscataway, NJ) for detection.

### Cell Growth and Mitotic Index

Stably transfected cells were seeded and counted after 6 days in culture using a hemacytometer in at least three independent experiments. Trypan blue exclusion was used to determine viable cells for counting. To determine mitotic index, cells were either stained with Hoechst 33258 or immunostained with anti-phospho-histone H3. Following the incubation with Cy3™-conjugated affinity purified secondary antibody (Jackson Laboratory, West Grove, PA), the coverslips were mounted using Antifade. At least 300~500 cells in 10 randomly selected fields were counted to determine mitotic figures or cells intensely labeled with anti-phosphorylated histone H3. Statistical analysis was performed using MS Excel and SigmaPlot (SPSS Science, Chicago, IL). Student *t*-tests or one-way ANOVA were conducted and *p*-values were assigned. The significance level was set at 0.05.

### Colony Formation in Soft Agar

A soft agar colony formation assay was performed using six-well plates. Each well contained 1% agar in 2× RPMI 1640 medium as the bottom layer and 0.6% agar in 2× RPMI 1640 medium as the top layer. 5,000 cells were plated into the top layer. The plates were incubated in a humidified incubator with 5% CO_2 _in air at 37°C. Colonies were stained with 0.04% Crystal violet and counted in 20 randomly selected fields. The number of colonies was plotted as the mean ± SD.

### Prostate Tumor Xenograft Growth in Nude Mice

Male, athymic nude (nu/nu) mice (Charles River Lab, Wilmington, MA) were obtained and used at 4–8 weeks of age. Animals were kept under pathogen free conditions according to the guidelines of East Carolina University Animal Use Protocol. Animals were given subcutaneous injections of 4 ×10^6 ^CWR22Rv-1 cells in RPMI 1640 medium plus cold Matrigel (BD Biosciences) or PC-3 cells in RPMI 1640 medium without Matrigel into the dorsal flank of nude mice. Once xenografts became established, their size was measured with a caliper once a week. The tumor volume was calculated by the formula: length × width × height × 0.5236 [37]. At the end of each experiment, animals were sacrificed and resulting final tumor volumes were determined.

### Histological Examination and Immunofluorescent Light Microscopy

Five-micrometer sections were prepared from the tumors in nude mice and placed onto charged glass slides. They were stained with Hematoxylin. For visualization of EGFP or EGFP-δ-catenin expressing cells in nude mice, 5 μm sections were prepared and fixed in 4% paraformaldehyde. They were analyzed under the Zeiss Axiovert inverted fluorescent light microscope equipped with MetaMorph imaging software.

### Protein Expression Analyzed by Western Blots

Cells were lysed in RIPA buffer with protease and phosphatase inhibitor cocktails, and subjected to protein solubilization and SDS-PAGE analysis. Proteins were western blotted using anti-Erk1/2 (1:1000), anti-p21^Cip1^, anti-survivin (1:1000), anti-histone H3 (1:1000), and anti-phosphorylated histone H3 (1:1000). Anti-actin (1:5000) was used as a loading control. Following appropriate secondary antibody incubations, the blots were developed using enhanced chemiluminescence.

### RT^2 ^Profiler™ PCR Array and Real-time PCR

CWR22Rv-1 cells with or without δ-catenin overexpression were re-plated in RPMI 1640 media supplemented with 10% FBS and 0.25% G418 at 37°C in a 5% CO2 atmosphere. After 2 days, the total RNA was isolated using RNeasy Mini kit (Qiagen). The single strand cDNA from 2–3 μg total RNA was synthesized using RT^2 ^first strand kit (SABioscience). Real-Time PCR was performed according to the User Manual of RT^2 ^Profiler PCR array system (SABioscience) using SYBR Green PCR Master Mix in an iCycler iQ Multicolor Detection System (Bio-Rad). Three selected PCR arrays, including pathways of apoptosis (Catalog No. PAHS-012A) and cell cycle (Catalog No. PAHS-020A) were repeated three times and the data were analyzed using Excel-based PCR Array Data Analysis Templates (SABioscience). Additional real-time PCR analyses were performed to compare RNA expression of *cyclin D1*, *Bcl2L1 *and *HK2 *between control vector transfected cells and that of δ-*catenin *overexpressing cells. For these experiments, the primer set for *cyclin D1 *was 5'-AGAAGCTGTGCATCTACACCGACA-3' (forward) and 5'-TGGAGGGCGGATTGGAAATGAACT-3' (reverse), the primer set for *BcL2L1 *was 5'-GTCGCATTGTGGCCTTTTTCTCC-3' (forward) and 5'-AGCTGCGATCCGACTCACCAATAC-3' (reverse), while the primer set for *HK2 *was 5'-GCCTTCGGGGACAATGGATGC-3' (forward) and 5'-TCTGCTTGCCGGGGTTGAGTG-3' (reverse).

## Competing interests

The authors declare that they have no competing interests.

## Authors' contributions

YZ performed most of the experiments and contributed to the data analyses. AA contributed to the cell viability measurements while JL performed PCR arrays. TW contributed to the mouse transplantation experiments. KK designed and subcloned δ-catenin truncation constructs. YHC and DMT contributed to the design of the experiments, and QL contributed to the design of the entire study and the editing of the manuscript.
